# Comparative Analysis of Phytochemical Profile and Antioxidant and Antimicrobial Activity of Green Extracts from *Quercus ilex* and *Quercus robur* Acorns

**DOI:** 10.3390/molecules31020277

**Published:** 2026-01-13

**Authors:** Diego Gonzalez-Iglesias, Francisco Martinez-Vazquez, Laura Rubio, Jesús María Vielba, Trinidad de Miguel, Marta Lores

**Affiliations:** 1 Laboratorio de Investigación y Desarrollo de Soluciones Analíticas, Department of Analytical Chemistry, Nutrition and Food Science, Faculty of Chemistry, Universidade de Santiago de Compostela, 15782 Santiago de Compostela, Spain; diegogonzalez.iglesias@usc.es (D.G.-I.); franciscojose.martinez@rai.usc.es (F.M.-V.); 2 i-Grape, Via Isaac Peral 32, 15890 Santiago de Compostela, Spain; laura.rubio@i-grape.es; 3 Misión Biológica de Galicia, Consejo Superior de Investigaciones Científicas, Avda. de Vigo s/n, 15705 Santiago de Compostela, Spain; jmvielba@mbg.csic.es; 4 Department of Microbiology and Parasitology, Faculty of Pharmacy, Universidade de Santiago de Compostela, 15782 Santiago de Compostela, Spain; trinidad.demiguel@usc.es

**Keywords:** *Quercus*, acorns, polyphenols, antioxidant activity, antimicrobial activity, green extraction, MSAT, UHPLC-QToF

## Abstract

An environmentally friendly extraction strategy based on an MSAT (Medium Scale Ambient Temperature) system was applied to *Quercus ilex* and *Quercus robur* acorns with the aim of maximizing polyphenolic yield and antioxidant activity while minimizing solvent consumption. Operational parameters were first optimized for *Quercus ilex* using a BBD-RSM (Box–Behnken response surface methodology), where the optimum working zone corresponds to the values of 200 g of acorn, 100 mL of extracting solvent, and 0.5 dispersant/acorn ratio. Subsequently, these conditions were applied to *Quercus robur* to enable an interspecific comparison. Extracts were evaluated in terms of total polyphenolic content, antioxidant activity, reducing sugars, proteins, targeted polyphenols quantified by UHPLC-QToF, and antimicrobial activity. Optimal extractions from *Quercus ilex* reached 25,072 mgGAE L^−1^ and 162 mmolTE L^−1^, while *Quercus robur* extracts showed markedly superior values of 35,822 mgGAE L^−1^ and 234 mmolTE L^−1^. Polyphenol quantification revealed higher concentrations of gallotannins in *Quercus robur* and procyanidins and catechin in *Quercus ilex*. The extracts showed strong antibacterial activity, especially *Quercus ilex* against *S. aureus* with a MIC ≤ 0.63%. Furthermore, it has been demonstrated for the first time that acorn extracts can inhibit the growth of *Phytophthora cinnamomi* in vitro, with *Quercus robur* extracts having a MIC ≤ 0.1% and *Quercus ilex* extracts ≤ 1%.

## 1. Introduction

*Quercus* is a genus within the Fagaceae family, comprising around 400 tree species whose fruits are acorns. This genus is widely distributed in the temperate regions of the northern hemisphere in Europe, western Asia and North America, and includes species such as the pedunculate oak (*Quercus robur* L.) and the holm oak (*Quercus ilex* L.). Due to their carbohydrate, amino acid, protein, lipid and various sterols content, acorns have long been used as a source of tannins, oil, and especially, food [1]. Acorns were an important ingredient in the past, as they were used to bake bread, in years of scarcity, but their consumption by humans has been almost completely lost. Currently, they are mainly used to feed animals, specifically the Iberian pig breed [2].

In addition to their use for food purposes, acorns of this genus have been used in traditional medicine as haemostatic, astringent, antimicrobial, and healing agents due to their anti-inflammatory, antioxidant, and antibacterial properties thanks to their content of phenolic acids, tannins, flavonoids, lignans, stilbenoids, coumarins, monoterpenes, triterpenes, and steroids. Indeed, most of the biological effects of these plants have been attributed to polyphenolic content [3,4,5,6].

Polyphenols possess an optimal chemical structure for the neutralization of oxygen radicals. They act as highly effective proton and electron donors and acceptors, enabling them to stabilize free radicals and participate in the chelation of transition metal ions, thereby inhibiting metal-catalyzed reactions such as the Fenton reaction. Common oak plants can synthesize a significant number of phenols in vegetative and generative organs, which are essential for the formation of systemic plant resistance [7]. In addition, *Quercus* polyphenols have been the subject of intense research due to their important role in the maturation of wines in oak barrels [8,9].

Currently, different processes are used to obtain bioactive extracts from acorns, with the drawbacks of low scalability combined with high solvent consumption, such as ultrasound-assisted extraction (UAE), microwave-assisted extraction (MAE) and deep eutectic solvents (DES), or high material and equipment costs, such as supercritical fluid extraction (SFE-CO_2_) [10,11,12,13]. On the other hand, the medium scale ambient temperature (MSAT) extraction technique, developed in our research group and protected under patent applications, is gaining interest for obtaining bioactive extracts from various organic matrices due to its proven scalability to industrial level, versatility, and easy combination with the use of solvents generally recognized as safe (GRAS) [14,15]. It has been applied to obtain extracts from different varieties of blueberries and white grape marc both on a laboratory scale and on a pilot scale with excellent yields [16].

Acorns’ bioactive compounds open a new path for exploring their potential anti-pathogenic activity. Activity of *Quercus* extracts against pathogenic bacteria and fungi has been proved [17]. This fact, together with the interest risen in recent decades, about finding natural bioactive compounds against different diseases due to antimicrobial resistance, among other issues, makes acorns a good candidate to find new effective treatments and therapeutic strategies.

Therefore, the objective of this study is to conduct a comprehensive analysis of the MSAT extraction procedure to obtain bioactive extracts from Galician *Quercus ilex* and *Quercus robur* acorns, as well as to carry out a comparison of their polyphenolic profile and antimicrobial activity, especially against *Phytophthora cinnamomi*, the main cause of decline of *Quercus* trees in Mediterranean regions. The operating parameters involved in the extraction process were optimized to maximize total polyphenolic content and antioxidant activity using a Box–Behnken design response surface methodology (BBD-RSM).

## 2. Results and Discussion

### 2.1. MSAT Extraction Operational Optimization

In an initial approach, an operational range was defined considering three independent variables: acorn mass, extract volume, and dispersant/acorn ratio, keeping the extracting solvent fixed as ethyl lactate/water (50:50, *v*/*v*) and SiO_2_ as disruptor, as shown in Table 1.

For the analysis of operational factors, a Box–Behnken design was used to provide a measure of operational stability throughout the tests. The sample size and the maximum capacity of the glass column were the two limitations that determined both the choice of optimization design and the levels of the operational parameters. Table 2 summarizes the design matrix comprising 15 trials bounded by twelve edge points and three centre points. The total polyphenolic content (TPC) and the antioxidant activity (AA) of the extracts were considered as the dependent variables to evaluate the results of the design.

TPC values showed clear differences among the design experiments. However, only two factors were statistically significant: the amount of acorn (A, *p* = 0.005), and the volume of extraction solvent (B, *p* = 0.002). Antioxidant activity followed the same behaviour as TPC with A (*p* = 0.001) and B (*p* < 0.001) being statistically significant. In contrast, the linear effect of C and all quadratic effects and interactions did not reach statistical significance for either TPC or AA.

**Figure 1:**
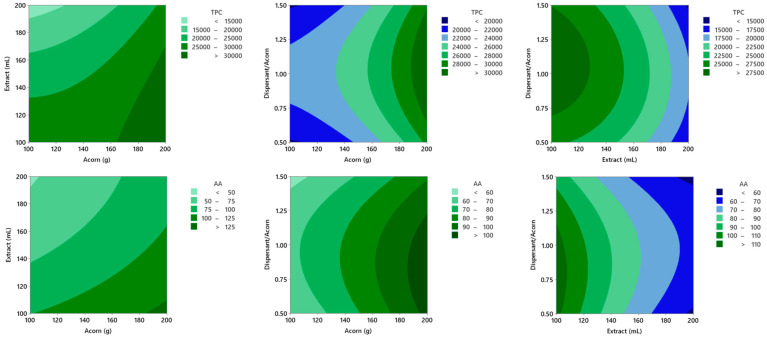
Response surfaces obtained from the Box–Behnken optimization design. The top row shows the response of the independent variable total polyphenolic content (TPC, mgGAE L^−1^) as a function of the combinations of the dependent variables, while the bottom row shows the antioxidant activity (AA, mmolTE L^−1^). The greener the response, the more bioactive the extract.

Figure 1 shows the response surface graphs obtained for TPC and AA, revealing a progressive positive trend for both factors as the mass of acorn increases and the volume of extraction solvent decreases. TPC values obtained from the design range from 13,094 to 31,680 mgGAE L^−1^, while AA values range from 47.9 to 118.0 mmolTE L^−1^. The lowest TPC values in the range obtained are still higher than those from pomegranate juices, widely known to be one of the fruits richest in polyphenols, where the best results range between 10,086 and 11,000 mgGAE L^−1^ [18,19]. Direct comparison of AA results with other studies using different extraction processes is limited because they tend to refer to sample weight (mmolTE g^−1^ dry weight) and do not specify reconstitution conditions, volumes, and densities in detail, which would require assumptions of dubious validity. However, it is possible to demonstrate the success resulting from scaling up the *Quercus ilex* acorn extraction process using the same solvent from matrix solid-phase dispersion (MSPD) to MSAT, where the TPC and AA values were 1110 mgGAE L^−1^ and 7.2 mmolTE L^−1^, respectively [20].

The criterion used to determine the optimal extraction conditions was to maximize TPC and AA. Therefore, the optimum working zone corresponds to the values of 200 g of acorn, 100 mL of extracting solvent and 0.5 dispersant/acorn ratio. At this point, the observed TPC value (25,072 mgGAE L^−1^) was lower than the predicted (29,394 mgGAE L^−1^), whereas the observed AA value (162 mmolTE L^−1^) was higher than the predicted (133 mmolTE L^−1^). Both the TPC and AA are above the expected 95% predictive range, with the TPC being lower than expected and the AA being higher. Although this behaviour is unusual, it has been described previously [21].

### 2.2. Solvent Proportion Selection

Once the operational parameters were optimized, a study was conducted to determine the ethyl lactate/water ratio (20, 35, 50, 65, and 80%) that maximizes the extract’s bioactivity. The analysis of the results includes the bioactive profile, measuring the TPC, AA, and EC_50_ (Half-Maximal Effective Concentration); the reducing sugar and protein content of the extracts; and the polyphenolic profile, quantifying 17 different polyphenols.

**Figure 2:**
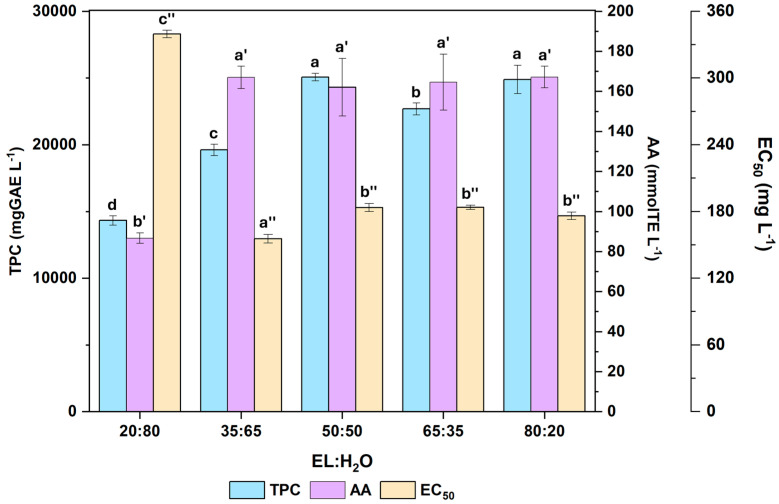
Total polyphenolic content (TPC), antioxidant activity (AA), and half-maximal effective concentration (EC_50_) of *Quercus ilex* acorn extracts using different ratios of ethyl lactate/water (EL:H_2_O) solvent. Mean value and standard deviation (x ± SD). For TPC n = 8, for AA and EC_50_ n = 3. The different letters for the same parameter denote a statistical difference with 95% confidence level between solvent ratios.

For each response variable of the antioxidant profile shown in Figure 2, a one-way ANOVA was performed. When the assumption of homoscedasticity was not met (TPC), Welch’s ANOVA was used followed by Games-Howell post hoc comparisons; when it was met (AA and EC_50_), post hoc comparisons were conducted using Tukey’s HSD test. For TPC, 50:50 and 80:20 ratios yielded the highest polyphenolic content (group a), followed by 65:35 (b), 35:65 (c), and 20:80 (d). Regarding AA, there were no significant differences among 80:20, 35:65, 65:35, and 50:50 (group a’), whereas the lowest ethyl lactate content (20:80) exhibited significantly lower activity (b’). For EC_50_, where lower values indicate higher potency, 35:65 ratio had the best EC_50_ (group a”), followed by 80:20, 50:50, and 65:35 (b”), while 20:80 was the least active (c”). Taken together, these results indicate that the 50:50 and 80:20 ratios provide the best overall balance, maximizing polyphenolic content and antioxidant activity while maintaining relatively low EC_50_. This behaviour, in which better extraction performance is obtained with isovolumetric mixtures of an organic solvent and water or with a higher proportion of the organic part, has already been demonstrated previously [16].

**Figure 3:**
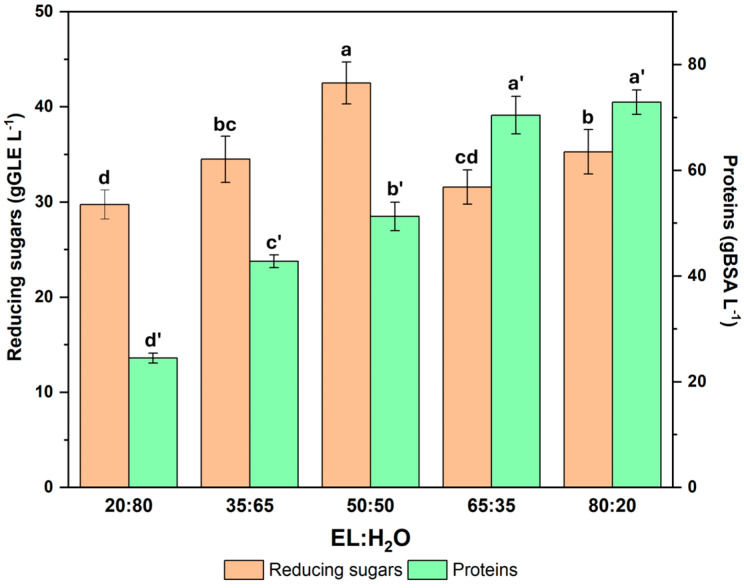
Reducing sugars and protein content of *Quercus ilex* acorn extracts using different ratios of ethyl lactate/water (EL:H_2_O) solvents. Mean value and standard deviation (x ± SD) (n = 8). The different letters for the same parameter denote a statistical difference with 95% confidence level between solvent ratios.

Figure 3 shows that the reducing power of the extracts is highest when the solvent mixture is isovolumetric, while the protein content increases as the organic part of the mixture increases. This is supported by the idea that the solvent/water synergy in intermediate mixtures better extracts polar/semi-polar molecules such as reducing sugars [22]. Furthermore, partition studies in two-phase systems with ethyl lactate confirm that the organic/water ratio is decisive in modulating selectivity towards polar compounds, as opposed to more hydrophobic macromolecules [23]. This reinforces the interpretation that a 50:50 ratio favours the solubilisation of reducing sugars, while a higher organic fraction increases protein recovery. However, it should also be considered that the DNS method may be overestimating the total amount of reducing sugars due to interference from polyphenol-rich extracts. In fact, both profiles are similar.

**Figure 4:**
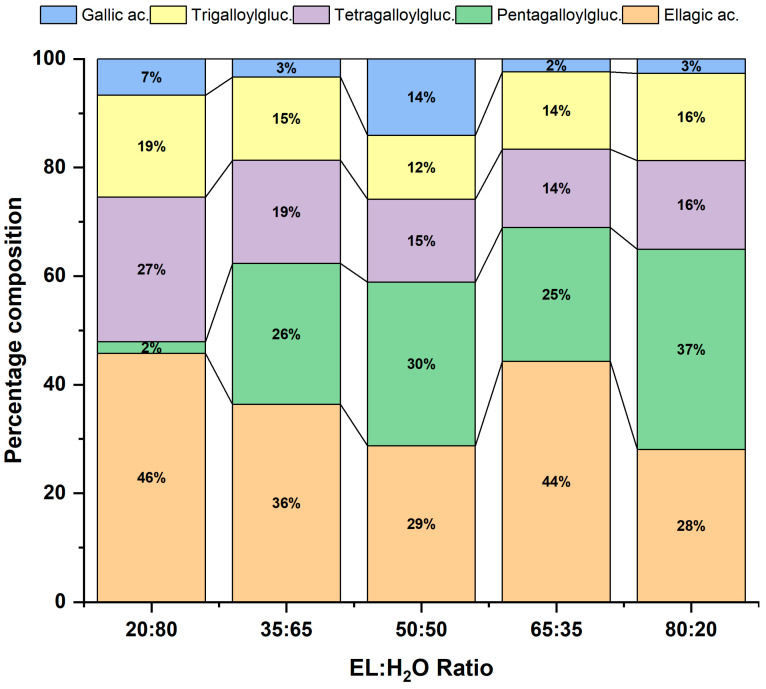
Main compounds of *Quercus ilex* acorn extracts using different ratios of ethyl lactate/water (EL:H_2_O) solvents quantified by UHPLC-QToF. Main compounds are gallic acid and ellagic acid as phenolic acids, and 1,3,6-trigalloylglucose, 1,2,3,6-tetragalloylglucose, and 1,2,3,4,6-pentagalloylglucose as gallotannins (hydrolysable tannins).

The main polyphenols present in acorns are phenolic acids and hydrolysable tannins, especially ellagitannins and gallotanins [17]. The results shown in Figure 4 corroborate this information for all solvent ratios. The main phenolic acids found were gallic acid and ellagic acid, while the main gallotannins were 1,3,6-trigalloylglucose, 1,2,3,6-tetragalloylglucose, and 1,2,3,4,6-pentagalloylglucose. The high content of gallic and ellagic acid is directly related to the content of hydrolysable tannins, as they are released by them [24]. Furthermore, based on the quantification of the different polyphenols, the 50:50 ratio proved to be superior to the 80:20 ratio with 3646 mg L^−1^ compared to 2889 mg L^−1^. This, combined with lower consumption of organic solvents and practical convenience, tips the balance in favour of the isovolumetric mixture. Complete information on the 17 polyphenols quantified by UHPLC-QToF in this solvent selection can be found in Supplementary Table S1.

### 2.3. Interspecific Comparison Between Quercus ilex and Quercus robur

Once the process parameters and solvent ratio that maximize the bioactivity of *Quercus ilex* acorn extracts had been optimized, they were applied to *Quercus robur* acorns. The results of this interspecific comparative study are shown in Table 3 and Table 4.

Extracts from *Quercus robur* acorns proved to be superior in all analyses reflecting bioactivity, obtaining very high values of 35,822 mgGAE L^−1^ and 234 mmolTE L^−1^, as well as a very low EC_50_ (DPPH) of 35 mg L^−1^. It should be noted that extracts from *Quercus robur* are 1.4 times higher in both total polyphenolic content and antioxidant activity, once again reflecting the direct correlation between them [25]. However, the reducing power and protein content show similar values for extracts from both species. This is consistent with what has been reported in the literature, as the ranges for sugars and proteins in different species tend to overlap despite the variability linked to the geographical origin and state of maturity of the acorns and the tree itself [26]. This could be because the essential components of primary metabolism, such as sugars and proteins, maintain a relatively conserved basal defence among species of the same genus, while secondary metabolites, such as polyphenols, have evolved specifically for defensive functions and tend to show greater variability among species [27,28].

As shown in Table 4, extracts from *Quercus robur* acorns, like those from *Quercus ilex* detailed above, are rich mainly in the same phenolic acids and gallotannins, as well as other polyphenols from the flavonoid family. As with the bioactivity results, a higher concentration of combined individual polyphenols has been quantified in *Quercus robur* extracts (8889 mg L^−1^) than in *Quercus ilex* extracts (3646 mg L^−1^). Most of these polyphenols have already been reported previously in acorns in different studies [17,29,30,31]. Others, such as β-glucogallin, which is the starter molecule in the biosynthetic pathway of all gallotannins, have only been identified in leaves, while epigallocatechin-3-O-gallate has been found in bark [32,33]. On the other hand, gallocatechin, which has only been quantified here in *Quercus robur* acorns, had already been identified previously, but only in bark [34].

**Figure 5:**
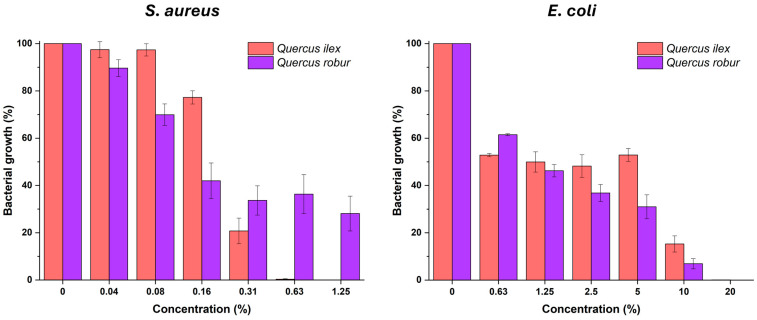
Inhibition of *S. aureus* and *E. coli* in vitro growth as a function of the concentration of *Quercus ilex* and *Quercus robur* acorn extracts. The positive control for bacterial growth corresponds to the columns with a concentration of 0%. Mean value and standard deviation (x ± SD) (n = 9).

Of the different polyphenols identified, the high concentration of gallic acid and ellagic acid present in both extracts should be highlighted. Furthermore, while *Quercus robur* extract has a higher abundance of gallotannins (more than double), *Quercus ilex* extract has a much higher concentration of procyanidins (six times more) and catechin (2.6 times more). These individual differences are more decisive when evaluating the antibacterial activity of the extracts than the total polyphenolic content. Figure 5 and Table 5 show the results of the inhibition of *S. aureus* and *E. coli* growth as a function of the concentration of the optimized extracts of *Quercus ilex* and *Quercus robur* acorns, as well as their minimum inhibitory concentration (MIC), inhibitory concentration 90% (IC_90_), and half-maximal inhibitory concentration (IC_50_).

Firstly, it should be noted that both graphs show plateau regions where bacterial growth remains stagnant at different concentrations. This is because the extracts are multicomponent and there is a balance between the adverse effects of polyphenols against bacteria and the nutritive or masking effects of the sugars and proteins present [35].

However, both extracts have good antibacterial activity against *S. aureus* and moderate activity against *E. coli*. It should be noted that *Quercus ilex* extract is far superior to *Quercus robur* extract when it comes to *S. aureus*, with an MIC of ≤0.63% and ≤2.5%, respectively. These results are because *Quercus ilex* acorns concentrate much more of the polyphenols with greater antibacterial potency against Gram-positive bacteria, procyanidins, and catechin, as they penetrate their cell wall better [36]. In contrast, against *E. coli*, both extracts require very high concentrations because the outer membrane prevents the entry of large polyphenols. In this case, the overall effect of the total gallotannin fraction predominates, which could slowly release gallic acid, explaining the slight superiority of *Quercus robur* in IC_90_ and IC_50_, although there is no statistical difference [37].

**Figure 6:**
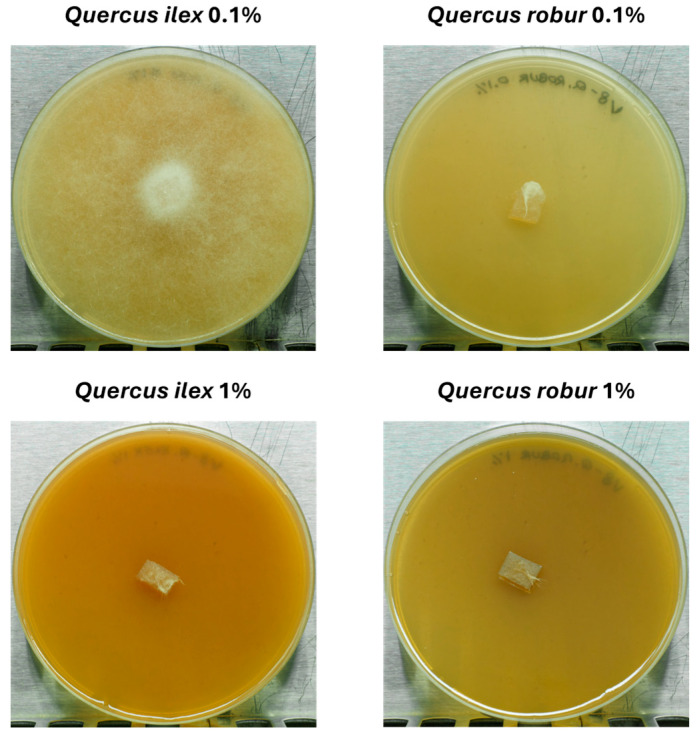
Inhibition of *Phytophthora cinnamomi* growth after 15 days of cultivation at 25 °C using extracts of *Quercus ilex* and *Quercus robur* acorns at 0.1% and 1% concentration. For the *Quercus ilex* 0.1% condition, there was growth across the entire plate, while in the other three conditions, inhibition was total.

To conclude this interspecific study between extracts of *Quercus ilex* and *Quercus robur* acorns, both extracts were tested against the oomycete *Phytophthora cinnamomi*, a root pathogen known to be consistently associated with tree decline and mortality of *Quercus* in the Mediterranean region. The results are shown below in Figure 6.

The results obtained are very revealing, as they demonstrate for the first time that acorns contain a chemical arsenal capable of inhibiting the growth of their main pathogen. On the plate treated with 0.1% *Quercus ilex* acorn extract, *Phytophthora cinnamomi* growth spread throughout the entire plate, while treatment with 1% *Quercus ilex* acorn extract and 0.1% and 1% *Quercus robur* acorn extract resulted in complete growth inhibition after 15 days of culture at 25 °C. Therefore, for *Quercus robur* extracts, the MIC is ≤0.1%, while for *Quercus ilex*, the MIC is ≤1%. This difference in behaviour between the two extracts may be due to the higher tannin content in *Querucs robur*, as its anti-oomycete activity has been demonstrated [38]. These results open the door to the use of these extracts in research aimed at their ultimate use in endotherapy against trees infected by *Phytophthora cinnamomi* or as a preventive treatment.

## 3. Materials and Methods

### 3.1. Standards and Reagents

The standards employed for the polyphenol quantification in the extracts and the bioactivities, with their suppliers, purity, and CAS numbers are summarized in Supplementary Table S2. The solvent used for the extraction process was ethyl lactate and ultrapure water MS-grade from Scharlab (Barcelona, Spain). Methanol MS-grade obtained by Sigma-Aldrich Chemie GmbH (Steinheim, Germany) and formic acid obtained by Merck (Darmstadt, Germany) were used for the mobile phase preparation in UHPLC-QToF, and 2-propanol obtained by Merck was used for calibrant.

### 3.2. Quercus Acorns

*Quercus ilex* and *Quercus robur* acorns with cupulas were manually collected from the area of O Courel, Galicia, Spain (42°37′40.0″ N 7°07′13.4″ W), and from the area of Narón, Galicia, Spain (43°29′43.4″ N 8°10′34.2″ W), respectively, during the month of September 2024. Five trees of each species were sampled. Acorns were properly identified and authenticated by Dr Francisco Javier Silva Pando (Department of Forest Ecosystems, Lourizán Forestry Research Centre, Xunta de Galicia, Galicia, Spain). The collected acorns were placed in food-grade bags (20 cm × 20 cm) hermetically sealed for freezing (−18 °C) to avoid oxidation. The moisture content was 59.8% for *Quercus ilex* acorns and 65.3% for *Quercus robur* acorns, measured in a moisture analyser ADAM PMB 202 (Adam Equipment, Milton Keynes, UK) using a temperature ramp up to 110 °C until stabilization.

### 3.3. Medium-Scale Ambient Temperature Extraction

Frozen acorns were weighed and crushed without pre-treatment in an electric blender until an average particle diameter of about 5 mm was obtained. Then, the acorns were dispersed with SiO_2_ (particle size 0.707 mm) using a mortar and pestle for 5 min. MSAT extraction was carried out on an Afora V-53721 glass column (23 cm × 50 mm Ø) with a 0-pore filter plate (160–250 mm) containing 1 g of SiO_2_ layer at the bottom. Then, the mixture of disrupted acorns and SiO_2_ was transferred to the column and compacted. Finally, the extract was eluted in different volumes, depending on the design, by adjusting the flow rate for a total extraction time of 60 min.

Ethyl lactate was selected as the solvent due to its recent success in extracting bioactive compounds from acorns and other plants, as well as its excellent affinity for them [39,40,41]. Furthermore, it is a GRAS solvent that can be obtained by synthesis or bio-based produced from renewable natural sources, and is environmentally friendly, as it can be completely degraded into CO_2_ and water [42]. The optimized liquid acorn extracts have been registered under the name Landranat^®^.

### 3.4. Total Polyphenolic Content

The Folin–Ciocalteu assay was used to determine the total polyphenolic content (TPC) of the *Quercus ilex* and *Quercus robur* acorns extracts following Zhang’s guidelines for microtitration in 96-well plates [43]. Briefly, 20 µL of diluted extract (with a dilution factor of 125 in MilliQ water) was mixed with 100 µL of Folin–Ciocalteu reagent (1:10, *v*/*v*) and 80 µL of sodium carbonate solution (7.5 g L^−1^). The mixture was homogenized and kept in the dark for 30 min. Then, the absorbance was measured at λ = 760 nm in a microplate reader SPECTROstar Nano (BMG LABTECH, Ortenberg, Germany). To express the TPC index, calibration curves of gallic acid covering a concentration range of 30–150 mg L^−1^ (0.200–0.800 absorbance units [AU]) were employed. TPC was expressed as milligrams of gallic acid equivalent per litre of extract (mgGAE L^−1^). This process is carried out in octuplicate.

### 3.5. Antioxidant Activity

To evaluate the antioxidant activity (AA) of the extracts as well as their half-maximal inhibitory concentration (EC_50_), a 2,2-diphenylyl-1-picrylhydrazyl radical (DPPH) assay was performed. To express the AA, a calibration curve of Trolox in the range of 3–31 mg L^−1^ (0.200–0.800 AU) was used. The AA was represented as millimoles Trolox equivalents per litre of extract (mmolTE L^−1^). The EC_50_ of the samples was also measured and referred to the quantity of acorns used to obtain the extract (mg L^−1^).

For the DPPH assay, the guidelines described by Symes were followed [44]. Briefly, 100 µL of each extract at eight different concentration levels were placed in a 96-well plate and mixed with 100 µL of DPPH reagent prepared in methanol. A control consisting of 100 µL of MilliQ water with 100 µL of DPPH reagent was also prepared. The mixture was kept in the dark for 10 min, and the measurement was performed at λ = 515 nm. This process is carried out in triplicate.

To calculate the antioxidant activity (mmolTE L^−1^), the absorbance of the sample was subtracted from the absorbance of the control, and this value was then substituted into the Trolox calibration curve. To calculate the EC50 (mg L^−1^), the radical scavenging (%) of eight serial dilutions was calculated using Equation (1) (DPPH radical scavenging (λ = 515 nm)), followed by linear interpolation between the two values flanking 50%.

(1) $$Radical\;scavenging\;(\%)=\frac{Abs\;of\;control-Abs\;of\;sample}{Abs\;of\;control}\times 100$$

### 3.6. Reducing Sugars

The total reducing sugar content of the extracts was determined by the 3,5-dinitrosalicylic acid (DNS) method following the scheme of Gonçalves et al. with slight modifications [45]. Briefly, 25 mL of the extract was mixed with 25 mL of DNS reagent. The reaction takes place in a bath at 100 °C for 5 min. The absorbance measurement was performed at λ = 540 nm. To express the total content of reducing sugars, a calibration curve of glucose was used in the range of 0.5–2 mg L^−1^ (0.200–0.800 AU). Results were represented as mg glucose equivalent per litre of extract (gGLE L^−1^). This process is carried out in octuplicate.

### 3.7. Proteins

The protein content in the extracts was determined using the bicinchoninic acid (BCA) method following Smith et al. guidelines, whereby 30 µL of extract is added to each well of a 96-well microplate and mixed with 240 µL of the working solution [46]. The working solution is prepared by mixing 50 parts of reagent A, consisting of a solution of 1% BCA, 0.4% NaOH, 0.95% NaHCO_3_, 2% Na_2_CO_3_, and 0.16% sodium tartrate, with 1 part of reagent B, which is a 4% CuSO_4_ solution. The reaction mixture is incubated at 37 °C in the spectrophotometer for 30 min and the absorbance is measured at λ = 562 nm. Protein content is expressed in grams of bovine serum albumin per litre of extract (gBSA L^−1^). This process is carried out in octuplicate.

### 3.8. UHPLC-QToF Analysis

The quantification of individual polyphenols in the extracts was carried out in an Elute UHPLC 1300 coupled to a quadrupole time-of-flight mass spectrometry (QToF) Compact Instrument (Bruker Daltonics, Mannheim, Germany). Column ThermoScientific (Darmstadt, Germany) HypersilGold aQ (1.9 mm, 100 mm × 2.1 mm) was kept at a constant temperature of 40 °C. Mobile phase consisted of 4 mM formic acid in water (A) and methanol (B). The total acquisition time was 20 min with a flow rate of 0.20 mL min^−1^. The elution gradient began with 5% (B) for 0.4 min (calibrant injection interval), followed by a gradual increase in phase B in the following phase and time intervals (%:min): 30:4.5, 37:8.0, 50:9.0, 90:11.0, 90:14.0, reaching the initial conditions at 16 min and maintaining them for 4 min, before the next run. In each acquisition, a calibrant was injected in a complementary manner to correct for mass deviation. This calibrant contained NaOH at a concentration of 1 mM in a 1:1 ratio of H_2_O:2-propanol and 0.2% formic acid.

Using an electrospray ionization (ESI) source and a bbCID acquisition mode with negative polarity, pseudomolecular ions [M-H]^−^ were mainly detected according to the method previously optimized by the research group. Compass HyStar and DataAnalysis version 5.1 (Build 201.2.4019) software were used for data acquisition and preprocessing, respectively. TASQ version 2024b was used for screening, confirming, and quantifying target polyphenols.

MS/MS table and chromatograms for the two species are included in Supplementary Table S3 and Supplementary Figures S1 and S2.

### 3.9. Antimicrobial Assays

Viability tests were performed following the recommendations of the EUCAST broth microdilution protocol; some adaptations were made for application to *S. aureus* and *E. coli* [47]. Briefly, in sterile 96-well plates different concentrations of extracts, depending on the bacteria (0%, 0.04%, 0.08%, 0.16%, 0.31%, 0.625%, 1.25%, and 2.5% *v/v* for *S. aureus* and 0%, 0.625%, 1.25%, 2.5%, 5%, 10%, and 20% *v/v* for *E. coli*), diluted and autoclaved in distilled water, were incubated with 5 × 10^6^ CFU mL^−1^ of bacteria in cation-supplemented Mueller–Hinton broth. Each well was buffered with 1M phosphate-buffered saline (PBS) at pH 7.4. Plates were incubated at 36 °C in a 5% CO_2_ atmosphere for 21 h. After incubation, 20 µL of a 0.5mg mL^−1^ resazurin solution (Sigma-Aldrich, St. Louis, MO, USA) was added to each well. The plates were left for incubation for 15 and 30 min, after which the fluorescence was measured using a FLUOR^®^ Star Omega plate reader (Ortenberg, Germany). The excitation and emission wavelengths were set to λ = 560 nm and λ = 590 nm, respectively. The data obtained from the fluorometer were used to calculate the IC_50_, IC_90_, and minimum inhibitory concentration (MIC). All assays were performed in triplicate.

*Phytophthora cinnamomi* was routinely grown in the lab in V8 agar medium (V8 juice 200 mL L^−1^, CaCO_3_ 2.8 g L^−1^, 15 g L^−1^ DIFCO agar, sterilized at 121 °C for 20 min) on 90 mm Petri dishes. For the extract tests, 7 mm × 5 mm agar plugs taken from the edge of actively growing cultures were used as inoculum and placed in the centre of V8 + extract containing Petri dishes, which were incubated in the dark at 25 ± 1 °C for 15 days [48].

### 3.10. Statistical Analysis

All data were expressed as mean ± standard deviation and all analyses were run in triplicate. The optimization of the extraction procedure was carried out by a Box–Behnken response surface methodology (BBD-RSM) evaluating the quadratic action of the considered factors and interactions. After confirming the homoscedasticity of the data, an analysis of variance (ANOVA) was carried out and Tukey’s HSD test was also performed on the experimental data using Minitab, LLC. 20.3. When the assumption of homoscedasticity was not met, Welch’s ANOVA was used followed by Games-Howell post hoc comparisons. All statistical operations were performed at a significance level of 5%. For antibacterial study, the extract inhibitory values, IC_50_ and IC_90_, were calculated by Graphpad Prism 9.0.

## 4. Conclusions

Optimization of the operational parameters of the MSAT methodology allowed the obtention of highly bioactive extracts from *Quercus ilex* and *Quercus robur* acorns, with a high polyphenol content and antioxidant activity. UHPLC-QToF analysis showed that both extracts are rich in phenolic acids, flavonoids, and gallotannins. *Quercus ilex* extract showed higher concentrations of gallic acid, catechin, procyanidins, isovanillic acid, 4-hydroxycinnamic acid, polydatin, and naringenin, while the *Quercus robur* extract was higher in gallotannins, ellagic acid, epicatechin gallate, and total quantity. The similarity in reducing sugar and protein content between species suggests that interspecific differences are mainly due to variations in secondary metabolites. In addition, both extracts showed high antibacterial activity, especially *Quercus ilex* against *S. aureus*. More importantly, it has been demonstrated for the first time that acorn extracts can inhibit the in vitro growth of the oomycete *Phytophthora cinnamomi*. These results demonstrate the MSAT extraction as an efficient, rapid, and green alternative for producing high-value extracts to develop functional ingredients. Potential applications range from animal nutrition and health to endotherapy for plant protection. Future research directions include an untargeted study (UHPLC-QToF) for in-depth chemical characterization of the extracts, toxicity studies, and semi-in vivo trials of plants infected with *Phytophthora cinnamomi*.

## Figures and Tables

**Table 1:** Levels of dependent variables used in the Box–Behnken response surface methodology optimization design.

Parameters	Legend	Low	High
Acorn (g)	A	100	200
Extracting solvent (mL)	B	100	200
Dispersant/acorn (g/g)	C	0.5	1.5

**Table 2:** Box–Behnken response surface methodology optimization design matrix. Type 0 are replicates at the design centre. Type 2 are points at the centre of the cube edges. Independent variables are A (acorn), B (extracting solvent), and C (dispersant/acorn). Dependent variables are total polyphenolic content (TPC) and antioxidant activity (AA).

Assay	Type	A (g)	B (mL)	C (g/g)	TPC (mg GAE L^−1^)	AA (mmol TE L^−1^)
1	2	100	100	1.0	30,151	97.8
2	2	200	100	1.5	31,680	109.8
3	2	100	200	1.0	13,094	47.9
4	2	200	200	1.0	24,457	92.6
5	2	100	150	0.5	19,217	56.0
6	2	200	150	0.5	29,722	97.4
7	2	100	150	1.5	18,313	61.8
8	2	200	150	1.5	30,585	92.8
9	2	150	100	0.5	24,219	118.0
10	2	150	200	0.5	15,812	61.8
11	2	150	100	1.5	27,262	92.3
12	2	150	200	1.5	15,709	52.0
13	0	150	150	1.0	23,635	88.4
14	0	150	150	1.0	28,740	76.8
15	0	150	150	1.0	23,418	88.8

**Table 3:** Total polyphenolic content (TPC), antioxidant activity (AA), half-maximal effective concentration (EC_50_), reducing sugars and proteins for MSAT extractions with the optimal operational parameters of *Quercus ilex* and *Quercus robur* acorns.

Acorns	TPC (mg GAE L^−1^)	AA (mmol TE L^−1^)	EC_50_ (mg L^−1^)	Reducing Sugars (g GLE L^−1^)	Proteins (g BSA L^−1^)
*Quercus ilex*	25,072 ± 278 ^b^	162 ± 14 ^b^	183 ± 4 ^b^	43 ± 2 ^a^	51 ± 3 ^a^
*Quercus robur*	35,822 ± 725 ^a^	234 ± 23 ^a^	95 ± 5 ^a^	35 ± 2 ^b^	54 ± 2 ^a^

Mean value and standard deviation (x ± SD). For TPC, reducing sugars and proteins (n = 8), for AA and EC_50_ n = 3. The different letters in a same column denote a statistical difference with 95% confidence level between *Quercus* species.

**Table 4:** Concentration expressed as mg L^−1^ of the polyphenols detected in *Quercus ilex* and *Quercus robur* acorn extracts and their retention time (RT).

	Compound	RT (Min)	*Quercus ilex*	*Quercus robur*
1	β-glucogallin	2.15	3.7 ± 0.2	1.3 ± 0.3
2	Gallic acid	3.55	505 ± 2	350 ± 20
3	Gallocatechin	4.33	n.d.	0.40 ± 0.02
4	Catechin	5.80	15.9 ± 0.8	6.0 ± 0.5
5	∑ Procyanidins B1 and B2	6.52	19.9 ± 0.4	3.9 ± 0.2
6	1,3,6-trigalloylglucose	6.62	422 ± 6	790 ± 70
7	Epigallocatechin-3-O-gallate	6.67	0.44 ± 0.01	0.48 ± 0.02
8	Isovanillic acid	6.80	4.4 ± 0.2	<LOQ
9	Procyanidin C1	6.96	5.9 ± 0.6	0.6 ± 0.1
10	4-hydroxycinnamic acid	7.11	0.27 ± 0.01	0.08 ± 0.02
11	∑ Procyanidin A1 and A2	7.25	0.24 ± 0.02	<LOQ
12	1,2,3,6-tetragalloylglucose	7.68	550 ± 20	1030 ± 70
13	Epicatechin-3-O-gallate	8.06	0.12 ± 0.01	0.48 ± 0.02
14	Polydatin	8.31	0.26 ± 0.02	0.08 ± 0.01
15	1,2,3,4,6-pentagalloylglucose	9.46	1080 ± 30	2400 ± 200
16	Quercetin-3-O-galactoside	9.93	0.13 ± 0.02	0.16 ± 0.03
17	Ellagic acid	11.46	1030 ± 10	4300 ± 200
18	Naringenin	12.03	8.0 ± 0.7	5.7 ± 0.7
	∑ Polyphenols		3646	8889

Mean value and standard deviation (x ± SD) (n = 3). Non-detected is expressed as n.d. Under limit of quantification is expressed as <LOQ.

**Table 5:** Minimum inhibitory concentration (MIC), inhibitory concentration 90% (IC_90_), and half-maximal inhibitory concentration (IC_50_) of acorns extracts from *Quercus ilex* and *Quercus robur* against *S. aureus* and *E. coli*.

	*S. aureus*		*E. coli*	
	*Quercus ilex*	*Quercus robur*	*Quercus ilex*	*Quercus robur*
MIC (%)	≤0.63 ^a^	≤2.5 ^b^	≤20 ^a^	≤20 ^a^
IC_90_ (%)	0.38 ± 0.04 ^a^	2.17± 0.05 ^b^	13.27 ± 1.43 ^a^	11.16 ± 2.03 ^a^
IC_50_ (%)	0.23 ± 0.02 ^b^	0.13 ± 0.02 ^a^	1.45 ± 0.30 ^a^	1.22 ± 0.20 ^a^

Mean value and standard deviation (x ± SD) (n = 9). The different letters for bacteria and parameters denote a statistical difference with 95% confidence level between *Quercus* species.

## Data Availability

All the data are available within the present manuscript and Supplementary Materials.

## Supplementary Materials

The following supporting information can be downloaded at: https://www.mdpi.com/article/10.3390/molecules31020277/s1, Table S1: Standards and reagents; Table S2: Concentration expressed as mg L^−1^ of the polyphenols detected in the analyzed species. Non-detected is expressed as n.d. Under limit of quantification is expressed as <LOQ. Table S3: Retention time (Rt), precursor ion, linear range and coefficients of determination (R^2^) for the quantified polyphenols in the acorn extracts. MS/MS fragmentation was performed by CID (Collision-Induced Dissociation) in the collision cell at 30 eV, with an additional in-source activation of 5 eV. Figure S1: Individual chromatograms, with retention time and molecular formula, of the different polyphenols quantified for the extracts of *Quercus ilex* acorns. Figure S2: Individual chromatograms, with retention time and molecular formula, of the different polyphenols quantified for the extracts of *Quercus robur* acorns.
