# Supplementary material for: Comparative Analysis of Phytochemical Profile and Antioxidant and Antimicrobial Activity of Green Extracts from Quercus ilex and Quercus robur Acorns
Source: Molecules. 2026 Jan 13;31(2):277. doi: 10.3390/molecules31020277 (PMC12843702; doi:10.3390/molecules31020277)
Supplement: Supplementary file 1 [file molecules-31-00277-s001.zip › molecules-4063796-supplementary.pdf]

Table S1. Concentration expressed as mg L^{-1} of the polyphenols detected in the analysed species. Non detected is expressed as n.d. Under limit of quantification is expressed as < LOQ.

	**Compound**	**RT (min)**	**LE:$H_2$0 (20:80)**	**LE:$H_2$0 (35:65)**	**LE:$H_2$0 (50:50)**	**LE:$H_2$0 (65:35)**	**LE:$H_2$0 (80:20)**
1	β-glucogallin	2.15	9.3 ± 0.6	7.5 ± 0.6	3.7 ± 0.2	6.7 ± 0.5	5.3 ± 0.2
2	Gallic acid	3.55	103 ± 9	91 ± 6	505 ± 2	78 ± 3	75 ± 2
3	Catechin	5.80	1.3 ± 0.1	1.7 ± 0.1	15.9 ± 0.8	6.4 ± 0.4	15.4 ± 0.7
4	∑ Procyanidins B1 and B2	6.52	4.23 ± 0.02	7.1 ± 0.2	19.9 ± 0.4	7.4 ± 0.7	18.3 ± 1
5	1,3,6-trigalloylglucose	6.62	288 ± 8	420 ± 20	422 ± 6	460 ± 20	460 ± 20
6	Epigallocatechin-3-O-gallate	6.67	<LOQ	<LOQ	0.44 ± 0.01	0.48 ± 0.05	0.56 ± 0.02
7	Isovanillic acid	6.80	6.7 ± 0.1	8.3 ± 0.1	4.4 ± 0.2	6.9 ± 0.2	7.1 ± 0.5
8	Procyanidin C1	6.96	1.62 ± 0.06	3.0 ± 0.1	5.9 ± 0.6	4.9 ± 0.2	6.5 ± 0.2
9	4-hydroxicynamic acid	7.11	0.65 ± 0.02	0.6 ± 0.1	0.27 ± 0.01	1.1 ± 0.1	0.74 ± 0.05
10	∑ Procyanidin A1 and A2	7.25	0.16 ± 0.03	0.24 ± 0.05	0.24 ± 0.02	0.15 ± 0.01	0.36 ± 0.02
11	1,2,3,6-tetragalloylglucose	7.68	410 ± 80	520 ± 20	550 ± 20	470 ± 10	464 ± 8
12	Epicatechin-3-O-gallate	8.06	<LOQ	<LOQ	0.12 ± 0.01	0.2 ± 0.1	0.42 ± 0.04
13	Polydatin	8.31	0.63 ± 0.01	0.43 ± 0.08	0.26 ± 0.02	0.8 ± 0.2	0.77 ± 0.04
14	1,2,3,4,6-pentagalloylglucose	9.46	33 ± 1	710 ± 40	1080 ± 30	800 [illegible]	1040 ± 20
15	Quercetin-3-O-galactoside	9.93	0.14 ± 0.01	0.08 ± 0.01	0.13 ± 0.02	0.04 ± 0.01	0.04 ± 0.01
16	Ellagic acid	11.46	700 ± 30	990 ± 70	1030 ± 10	1440 ± 90	790 ± 90
17	Naringenin	12.03	16.0 ± 0.6	6.8 ± 0.1	8.0 ± 0.7	4.7 ± 0.1	4.72 ± 0.02
	**∑ Polyphenols**		1575	2767	3646	3288	2889

Table S2. Standards and reagents.

Name	Formula	Purity (%)	Company	CAS
	Polyphenols			
β-glucogallin	$C_{13}H_{16}O_{10}$	95.1	[a]SIGMA	13405-60-2
Gallic acid	$C_7H_6O_5$	99.9	[a]SIGMA	149-91-7
Gallocatechin	$C_{15}H_{14}O_7$	99.9	[b]PURIFY	970-73-0
Catechin	$C_{15}H_{14}O_6$	98.0	[a]SIGMA	18829-70-4
Procyanidin B1	$C_{30}H_{26}O_{12}$	96.7	[c]EXTRAS	20315-25-7
Procyanidin B2	$C_{30}H_{26}O_{12}$	98.5	[c]EXTRAS	29106-49-8
1,3,6-trigalloylglucose	$C_{27}H_{24}O_{18}$	97.5	[b]PURIFY	18483-17-5
Epigallocatechin-3-O-gallate	$C_{22}H_{18}O_{11}$	99.3	[d]GLEN	989-51-5
Isovanillic acid	$C_8H_8O_4$	99.4	[a]SIGMA	645-09-9
Procyanidin C1	$C_{45}H_{38}O_{18}$	99.8	[b]PURIFY	37064-30-5
4-hydroxicynamic acid	$C_9H_8O_3$	99.9	[a]SIGMA	501-98-4
Procyanidin A1	$C_{30}H_{24}O_{12}$	98.8	[b]PURIFY	103883-03-0
Procyanidin A2	$C_{30}H_{24}O_{12}$	99.9	[a]SIGMA	41743-41-3
1,2,3,6-tetragalloylglucose	$C_{34}H_{28}O_{22}$	98.0	[b]PURIFY	79886-50-3
Epicatechin-3-O-gallate	$C_{22}H_{18}O_{10}$	98.0	[a]SIGMA	1257-08-5
Polydatin	$C_{20}H_{22}O_8$	99.0	[a]SIGMA	65914-17-2
1,2,3,4,6-pentagalloylglucose	$C_{41}H_{32}O_{26}$	99.0	[b]PURIFY	14937-32-7
Quercetin-3-O-galactoside	$C_{21}H_{20}O_{12}$	98.5	[d]GLEN	482-36-0
Ellagic acid	$C_{14}H_6O_8$	99.9	[a]SIGMA	476-66-4
Naringenin	$C_{15}H_{12}O_5$	99.5	[e]TARGET	480-41-1
	Other standards and reagents			
Folin reagent	$C_{10}H_5NaO_5S$	-	[a]SIGMA	521-24-4
DPPH	$C_{18}H_{12}N_5O_6$	99.2	[f]TCI	1898-66-4
Trolox	$C_{14}H_{18}O_4$	98.5	[a]SIGMA	53188-07-1
Sodium carbonate	Na_2CO_3	99.7	[g]PANREAC	497-19-8
Sodium hydrogen carbonate	$NaHCO_3$	99.9	[d]GLEN	144-55-8
Sodium hydroxide	NaOH	99.2	[a]SIGMA	1310-73-2
Sodium tartrate	$C_4H_4Na_2O_6{\cdot}2H_2O$	99.9	[d]GLEN	6106-24-7
Copper(II) sulfate	$CuSO_4$	99.3	[d]GLEN	7758-98-7
BCA	$C_{20}H_{10}N_2Na_2O_4$	95.0	[h]FCHEM	979-88-4
BSA	-	98.0	[d]GLEN	9048-46-8
DNS	$C_7H_4N_2O_7$	99.9	[i]THERMO	609-99-4
Potassium sodium tartrate tetrahydrate	$C_4H_4KNaO_6{\cdot}4H_2O$	99.9	[d]GLEN	6381-59-5
Glucose	$C_6H_{12}O_6$	99.3	[f]TCI	50-99-7

[a]Sigma Aldrich GmbH (Steinheim, Germany), [b]Biopurify Phytochemicals (Chengdu, China), [c]Extrasynthese (Genay, France), [d]Glentham Life Sciences (Corsham, United Kingdom), [e]Targetmol (Boston, United States of America), [f]TCI (Tokyo Chemical Industry) (Tokyo, Japan), [g]Panreac AppliChem (Barcelona, Spain), [h]Fluorochem (Hadfield, United Kingdom), [i]Thermo Fisher Scientific (Waltham, United States of America).

Table S3. Retention time (Rt), precursor ion, linear range and coefficients of determination (R^2) for the quantified polyphenols in the acorn extracts. MS/MS fragmentation was performed by CID (Collision-Induced Dissociation) in the collision cell at 30 eV, with an additional in-source activation of 5 eV.

Polyphenols	RT (min)	Formula	Precursor ion (m/z)	Linear range (mg L^{-1})	R^2
β-glucogallin	1.49	$C_{13}H_{16}O_{10}$	331.066	0.1-5	0.9959
Gallic acid	3.54	$C_7H_6O_5$	169.014	0.2-5	0.9852
Gallocatechin	4.33	$C_{15}H_{14}O_7$	305.067	0.1-10	0.9998
Catechin	5.81	$C_{15}H_{14}O_6$	289.072	0.2-5	0.9980
Procyanidins B2	6.08	$C_{30}H_{26}O_{12}$	577.135	0.2-5	0.9992
1,3,6-trigalloylglucose	6.82	$C_{30}H_{26}O_{12}$	635.088	0.1-10	0.9929
Epigallocatechin-3-O-gallate	6.69	$C_{27}H_{24}O_{18}$	457.078	0.2-5	0.9916
Isovanillic acid	7.10	$C_{22}H_{18}O_{11}$	167.035	0.5-10	0.9992
Procyanidin C1	6.55	$C_8H_8O_4$	865.199	0.2-5	0.9996
4-hydroxycinnamic acid	7.11	$C_{45}H_{38}O_{18}$	163.040	0.1-10	0.9999
Procyanidin A1	7.05	$C_9H_8O_3$	575.120	0.1-10	0.9995
1,2,3,6-tetragalloylglucose	7.81	$C_{30}H_{24}O_{12}$	787.099	0.1-10	0.9995
Epicatechin-3-O-gallate	7.99	$C_{30}H_{24}O_{12}$	441.083	0.2-5	0.9945
Polydatin	8.38	$C_{34}H_{28}O_{22}$	390.132	0.1-10	0.9994
1,2,3,4,6-pentagalloylglucose	9.33	$C_{22}H_{18}O_{10}$	939.110	0.1-10	0.9920
Quercetin-3-O-galactoside	9.93	$C_{20}H_{22}O_8$	463.088	0.1-10	0.9956
Ellagic acid	10.88	$C_{41}H_{32}O_{26}$	939.110	0.1-10	0.9946
Naringenin	11.96	$C_{21}H_{20}O_{12}$	271.060	0.1-10	0.9974

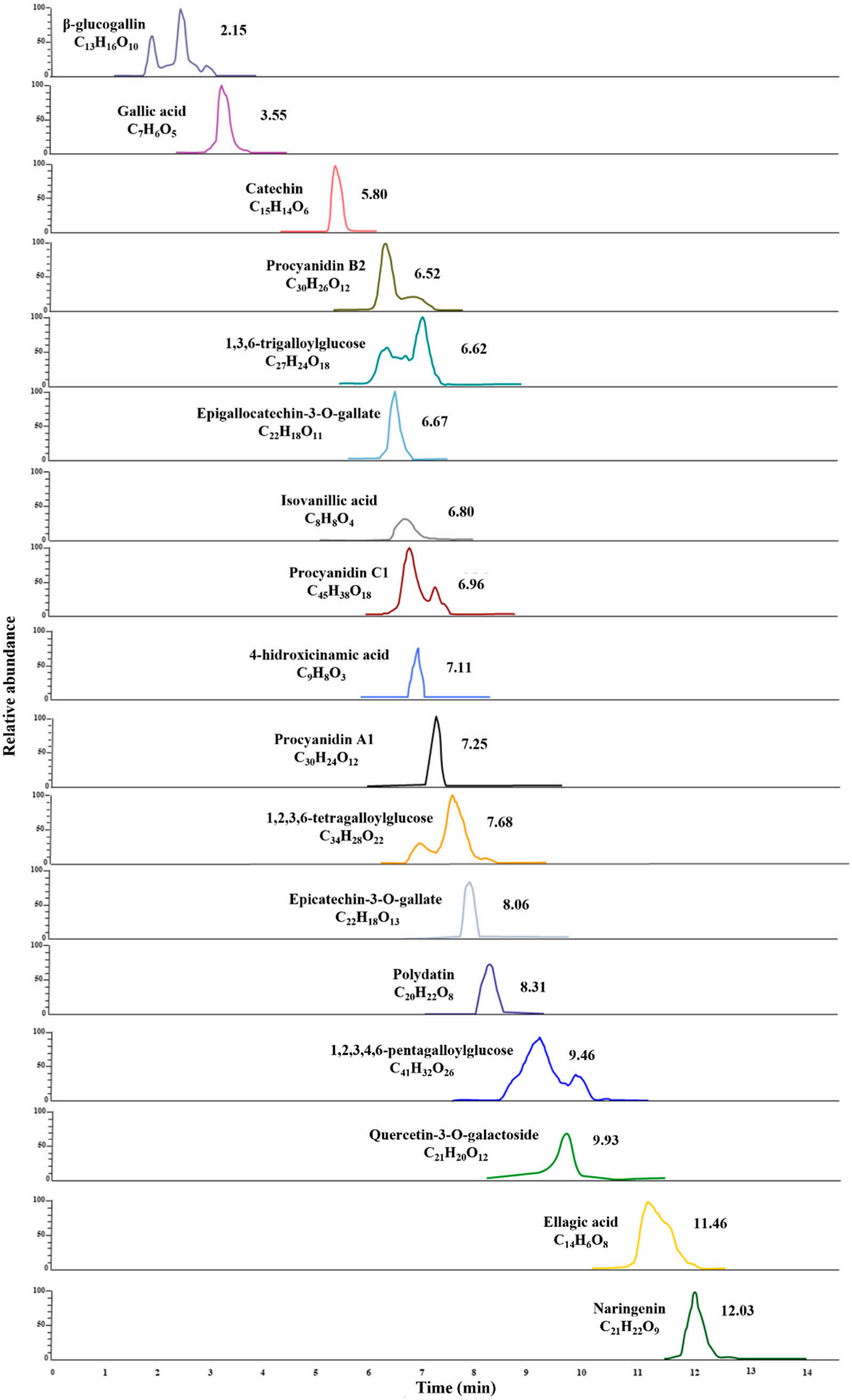

Figure S1. Individual chromatograms, with retention time and molecular formula, of the different polyphenols quantified for the extracts of *Quercus ilex* acorns.

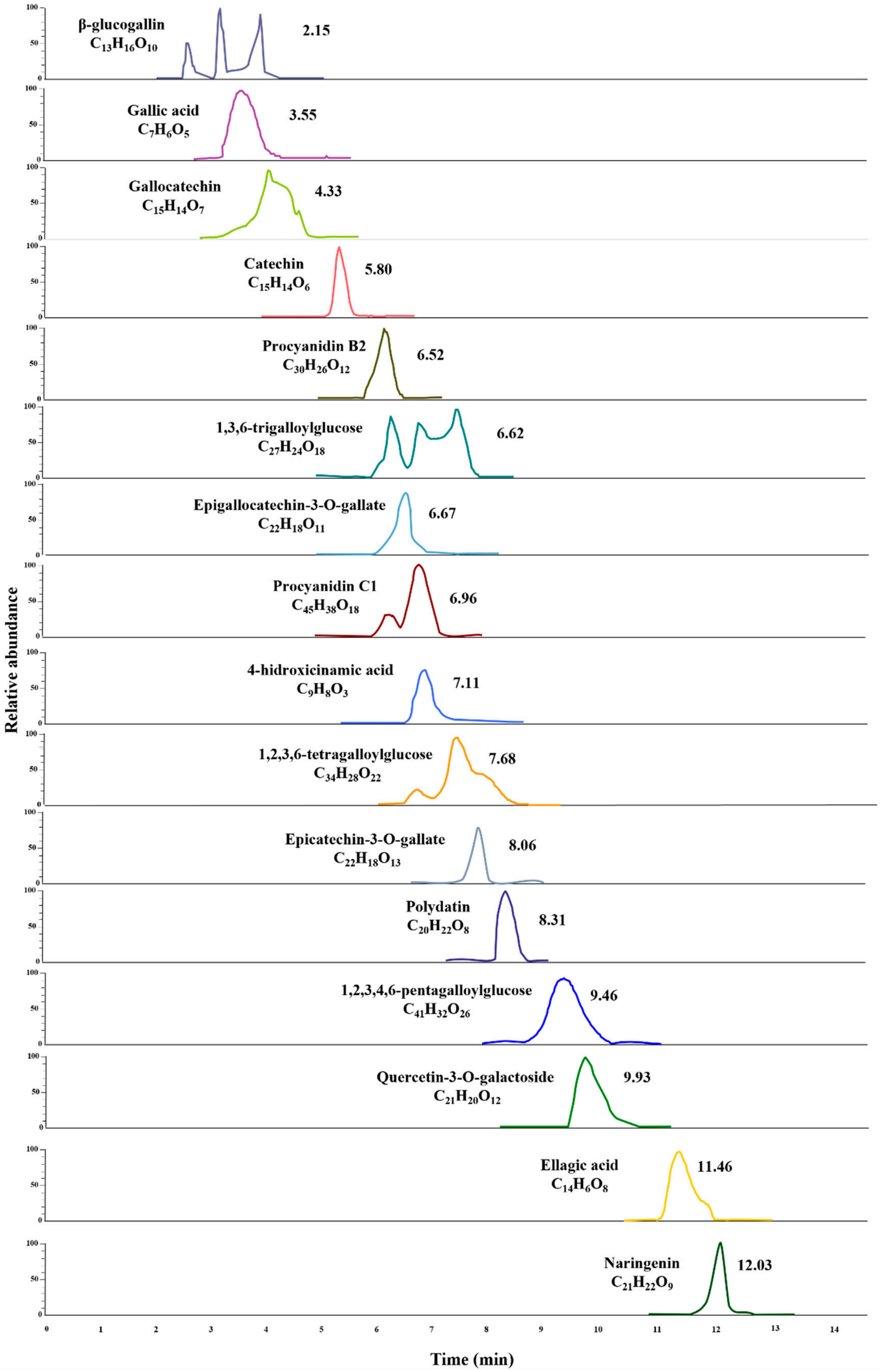

Figure S2. Individual chromatograms, with retention time and molecular formula, of the different polyphenols quantified for the extracts of *Quercus robur* acorns.
